# Nanobody-drug conjugates for targeting specific GPCR pairs

**DOI:** 10.1371/journal.pbio.3003483

**Published:** 2025-11-21

**Authors:** Xianglin Huang, Bryan L. Roth

**Affiliations:** Department of Pharmacology, School of Medicine, University of North Carolina at Chapel Hill, Chapel Hill, North Carolina, United States of America

## Abstract

Bitopic ligands that engage two distinct binding sites offer exciting opportunities for finely tuned control of G protein-coupled receptor signaling. This Primer explores a recent study in PLOS Biology that reports novel nanobody-small molecule conjugates and demonstrates their logic-gated activity at co-expressed receptor pairs with improved signaling profiles.

## Teaser

Bitopic ligands that engage two distinct binding sites offer exciting opportunities for finely tuned control of G protein-coupled receptor signaling. A recent study in *PLOS Biology* employed click chemistry to generate novel nanobody-small molecule conjugates and demonstrated their logic-gated activity at co-expressed receptor pairs with improved signaling profiles.

With their central roles in regulating diverse physiological responses, G protein-coupled receptors (GPCRs) represent one of the most important and attractive targets in drug discovery. Over the past decades, extensive research has been conducted to develop small-molecule and peptide therapeutics to modulate GPCR signaling. However, achieving high receptor selectivity and tissue specificity remains challenging due to the structural similarities and the widespread expression patterns of GPCRs. More recently, the development of GPCR-targeting nanobodies (Nbs), the variable fragments of camelid heavy chain-only antibodies, has drawn significant attention given its potentially ultra-high affinity and specificity [1]. In this study, the authors presented a strategy to tether a small-molecule drug and a Nb, creating a bitopic conjugate that harnesses the strengths of both therapeutic modalities [2]. While conceptually related to antibody-drug conjugate, which has been extensively investigated in cancer treatment [3], Nb-drug conjugate introduces unique implications in GPCR drug discovery with the design of logic-gated signaling implemented.

Starting from a well-studied class A GPCR, adenosine 2A receptor (A_2A_R), the authors conjugated a small-molecule A_2A_R agonist CGS21680(CGS) with epitope-targeting Nbs such as Nb_6E_ and Nb_ALFA_ using a method previously developed by their group [4]. Sortase-mediated labeling introduced a dibenzylcyclocytne probe at the N-terminus of Nb, which was covalently linked to azide-labeled CGS through click chemistry. Similarly, CGS-epitope conjugates were synthesized by coupling CGS-alkyne and azide-labeled 6E as previously described [5]. A luciferase-based cyclic adenosine monophosphate (cAMP) assay was used to quantify the activity of these CGS-Nb and CGS-epitope conjugates at wild-type (WT) A_2A_R and A_2A_R engineered with epitope tags 6E/ALFA at the N-terminus. While unconjugated CGS activated both WT and engineered A_2A_R, CGS-Nb and CGS-epitope showed little activity when their corresponding partner in each Nb-epitope pair is absent, confirming specificity. Remarkably, with the correct Nb-epitope interaction, Nb/epitope-conjugated CGS displayed >25-fold higher potency and more sustained signaling duration than the unconjugated CGS. An orthogonal G protein activation assay has also shown the enhanced potency of conjugated CGS. Importantly, under the conditions of low receptor expression, Nb-conjugated CGS maintained high potency and efficacy, whereas unconjugated or epitope-conjugated Nb showed minimal activity except at high concentrations. These findings highlight the advantage of Nb-conjugation in facilitating ligand binding, potentially through increasing the local concentration of ligand, which could have significant implications, especially in physiological contexts where the level of receptor expression is limited.

**Fig 1:**
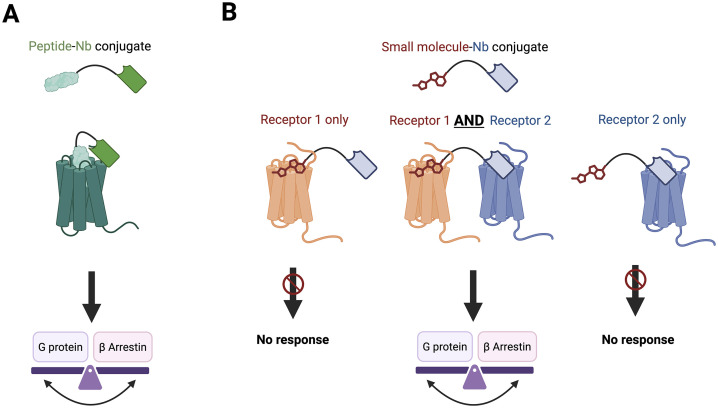
Illustration of the use of nanobody (Nb)-drug conjugates in tuning the activity of a single GPCR or a pair of GPCRs. **(A)** A peptide and a Nb targeting the same GPCR are conjugated and bind to both the orthosteric and allosteric sites of the same receptor, regulating the downstream responses. **(B)** The bitopic conjugate comprising a small molecule (targeting receptor 1) and a Nb (targeting receptor 2) only shows activity when both receptor 1 and receptor 2 are co-expressed. This dual-receptor targeting conjugate could provide more prolonged activity with stronger potency, alteration in biased signaling as well as potential tissue-specific pharmacology. The figure was created in BioRender.com.

Since engineered (Nb-/epitope-tagged) receptors do not exist naturally in vivo, the use of tandem epitope-Nb pairs primarily serves as a proof-of-concept demonstration of the advantages in developing Nb-small molecule conjugate for a single GPCR target. Similar strategies have been previously employed for generating Nb_6E_-peptide conjugate targeting angiotensin II type 1 receptor [6]. When receptor-specific Nbs are available, such as those developed for parathyroid hormone receptor-1 (PTHR1), a bitopic Nb-ligand conjugate generated can selectively bind to and engage two different sites of the same receptor, regulating the conformation changes [7,8] (Fig 1A). However, the development of Nb that binds extracellularly to class A GPCR is more challenging due to their smaller extracellular domains. The generation of therapeutically beneficial Nb-small molecule conjugates for a single receptor is therefore bottlenecked by the availability of Nbs. Then, what else could we use this exciting conjugating method to explore?

The authors next proposed an innovative application of their platform—using the chemical synthesis method to conjugate a small molecule with a Nb targeting another GPCR instead of epitope tags, therefore creating conjugates selectively active only in cells co-expressing both receptors. Another Gs-coupled receptor, PTHR1, was chosen as the first partner for A_2A_R, given the availability of Nb_PTHR1_. As shown in the study, CGS-Nb_PTHR1_ only shows activity when both A_2A_R and PTHR1 are co-expressed in a single cell population, demonstrating clear “AND” logic-gated activity. Both efficacy and potency of cAMP response vary depending on the expression ratio of the two receptors as well as the length of the linker between Nb_PTHR1_ and CGS. Similarly, a Nb-drug conjugate targeting A_2A_R co-expressed with glucagon-like peptide-1 receptor was synthesized and again showed “AND”-gated signaling. These together illustrate the tunability and adaptability of this platform in selectively activating A_2A_R only in the presence of specific receptor partners.

Given the lack of β-arrestin recruitment of A_2A_R, another pair of receptor partners, PTHR1 and G_i_ protein-coupled μ-opioid receptor (MOR), was chosen to investigate the potential of dual-targeting Nb-drug conjugates in regulating the signaling bias between G proteins and β-arrestins. They conjugated the MOR agonist DynA8 with Nb_PTHR1_, creating a biopic conjugate that was significantly more potent than DynA8 alone in both G_i2_ activation and β-arrestin2 recruitment. Consistent with the conjugate targeting A_2A_R-PTHR1 pair, the DynA8-Nb_PTHR1_ conjugate was only active when both receptors were co-expressed, and the potency increased with higher PTHR1 levels. Interestingly, the improved potency of DynA8-Nb_PTHR1_ in β-arrestin recruitment was higher (12.5-fold) than in G_i2_ protein activation (4.8-fold), making the conjugate less G-protein biased when compared with DynA8 alone. These findings illustrate the potentials of bitopic conjugates to tune signaling bias that differs from the pharmacological profile of the small molecule alone. With suitable combinations of the receptor partners and the choice of small molecules, highly potent, pathway-specific and tissue-specific biased signaling could be achieved for effective therapeutic benefits via the minimization of side effects (Fig 1B).

In summary, the study by Sachdev and colleagues [2] illuminates a new avenue for tuning biased signaling in a tissue-specific manner by tethering effective small-molecule drugs with ultra-selective Nbs for targeting GPCR pairs. With the enriched pharmacological knowledge of small molecules accumulated over decades, the rapid accelerations in the discovery of extracellularly binding GPCR Nb [9] aided by powerful *in silico* tools [10], combining with the growing insights in tissue-specific distribution landscapes of GPCRs [11], the availability of this conjugating strategy and its dual-acting “AND”-gated design reveals an exciting opportunity for the future of GPCR drug discovery.

## Abbreviations

cAMP: cyclic adenosine monophosphate
GPCRs: G protein-coupled receptors
MOR: μ-opioid receptor
PTHR1: parathyroid hormone receptor-1
WT: wild-type
